# Positron Emission Tomography/Computed Tomography in Bladder Cancer: The Role of [^18^F]FDG and Non-FDG Radiotracers

**DOI:** 10.3390/medicina62040703

**Published:** 2026-04-07

**Authors:** Hanna Falińska, Ewa Witkowska-Patena, Karolina Krzyżanowska, Mirosław Dziuk

**Affiliations:** 1 Military Institute of Medicine—National Research Institute, Department of Nuclear Medicine, Szaserów 128, 04-141 Warsaw, Polandmdziuk@wim.mil.pl (M.D.); 2 Affidea Poland, Plac Europejski 2, 00-844 Warsaw, Poland

**Keywords:** bladder cancer, [^18^F]FDG PET/CT, FAPI, PET/CT

## Abstract

*Background and Objectives*: Bladder cancer is one of the most common malignancies of the urinary tract and poses a significant clinical challenge due to its biological heterogeneity and high rates of recurrence and progression. Urothelial carcinoma represents the predominant histological subtype, ranging from non-muscle-invasive disease with relatively favorable outcomes to aggressive muscle-invasive and metastatic forms associated with poor prognosis. Accurate diagnosis, staging, prognostic stratification, and assessment of treatment response are therefore essential for optimal patient management. The objective of this review is to summarize and critically evaluate the current evidence on the role of positron emission tomography/computed tomography (PET/CT) in bladder cancer, with particular emphasis on [^18^F]FDG PET/CT and non-FDG radiotracers. *Materials and Methods*: A narrative review of the available literature was performed, focusing on clinical studies, review articles, and guideline documents addressing the use of PET/CT in bladder cancer. The literature search included articles published between 2000 and 2025, while earlier landmark studies were selectively included if considered historically important for understanding the development of PET/CT imaging in bladder cancer. The initial search yielded over 500 records; after screening titles and abstracts, more than 100 articles were selected for full-text evaluation. The analyzed evidence encompasses the clinical applications of [^18^F]FDG PET/CT and alternative radiotracers, including choline-, acetate-, methionine-, and sodium fluoride-based tracers, and fibroblast activation protein inhibitors (FAPI), across different stages of disease and clinical settings. *Results*: Conventional imaging modalities, such as computed tomography and magnetic resonance imaging, provide important anatomical information but remain limited in the evaluation of lymph node involvement, early metastatic disease, treatment response, and disease recurrence. Despite limitations related to physiological urinary excretion, [^18^F]FDG PET/CT has demonstrated clinical value in selected scenarios, particularly for staging, prognostic assessment, detection of recurrence, and response evaluation. To overcome FDG-related constraints, several non-FDG radiotracers have been investigated. Among these, FAPI PET/CT has emerged as a promising modality due to its ability to target the tumor stroma, potentially improving lesion detectability and tumor-to-background contrast. *Conclusions*: This review summarizes and critically evaluates current evidence on the role of PET/CT in bladder cancer, with a focus on [^18^F]FDG PET/CT and non-FDG radiotracers. The discussed studies highlight their applications in primary diagnosis, staging, prognostic assessment, detection of recurrence, and evaluation of treatment response, as well as their respective advantages and limitations. Furthermore, potential future directions for PET/CT imaging in clinical practice are outlined, emphasizing the need for further research to clarify the optimal use of established and emerging radiotracers.

## 1. Introduction

Bladder cancer remains a common and clinically challenging malignancy due to its heterogeneous behavior, high recurrence rates, and potential for early metastatic spread [1]. While cystoscopy and morphological imaging remain central to diagnosis and staging, their ability to accurately assess nodal involvement, distant metastases, and treatment response can be limited. Molecular imaging with positron emission tomography/computed tomography (PET/CT), using both [^18^F]FDG and novel non-FDG radiotracers, has therefore emerged as a valuable adjunct in the diagnostic workup of bladder cancer.

In clinical practice, PET/CT may serve different purposes depending on the disease setting, including initial staging of muscle-invasive bladder cancer, detection of nodal and distant metastases, assessment of treatment response, prognostic stratification, and evaluation of suspected recurrence. Its potential impact therefore varies across the diagnostic and therapeutic pathway.

The theoretical rationale for PET/CT in bladder cancer is based on the ability to visualize distinct biological processes beyond anatomical changes. The glucose analogue [^18^F]FDG reflects increased glycolytic activity of tumor cells, which is particularly relevant in aggressive and metastatic disease. However, intense physiological urinary excretion limits its performance in assessing the primary tumor. To address these limitations, alternative tracers targeting different metabolic or microenvironmental pathways have been investigated.

Choline tracers reflect membrane lipid synthesis, acetate is a marker of lipid metabolism and fatty acid synthesis, methionine is associated with amino acid transport and protein synthesis, sodium fluoride targets osteoblastic activity in skeletal metastases, and fibroblast activation protein inhibitor (FAPI) tracers visualize tumor-associated fibroblasts within the stromal compartment.

By targeting complementary biological mechanisms—tumor metabolism, membrane turnover, amino acid transport, bone remodeling, and stromal activation—these radiotracers provide a broader molecular perspective on bladder cancer biology. Understanding these distinct mechanisms is essential for defining the appropriate clinical role and limitations of PET/CT across different stages of the disease.

## 2. Materials and Methods

This narrative review was conducted to summarize and critically evaluate the current evidence regarding the role of positron emission tomography/computed tomography (PET/CT) in bladder cancer. A literature search was performed using electronic databases, particularly PubMed/MEDLINE. Additionally, the reference lists of relevant review articles and guideline documents were manually screened to identify further pertinent publications. The search included articles published from 2000 to 2025. Earlier landmark studies were included selectively if considered historically important for understanding the development of PET/CT imaging in bladder cancer. The following key search terms were used: “bladder cancer” OR “urothelial carcinoma”; “PET/CT” OR “positron emission tomography”; “[^18^F]FDG” OR “fluorodeoxyglucose”; “FAPI” OR “fibroblast activation protein inhibitor”; “choline”; “acetate”; “methionine”; “sodium fluoride”. Boolean operators (AND, OR) were used to combine terms appropriately. The inclusion criteria comprised original clinical research studies (both prospective and retrospective), meta-analyses, systematic reviews, guideline documents, and expert consensus statements, as well as studies evaluating the role of PET/CT in bladder cancer, including its application in diagnosis, staging, prognostic assessment, detection of recurrence, and treatment response evaluation. Only articles published in English were considered. The exclusion criteria included case reports and very small case series (<5 patients), animal studies, non-English publications, and studies not relevant to bladder cancer. Data extraction was performed in a descriptive manner, focusing on the type of radiotracer used, study design, clinical indications, and main findings.

This is a narrative review; all the sensitivities and specificities presented in the manuscript refer to values reported in individual studies or previously published meta-analyses, which are appropriately cited.

Images included in the figures and their corresponding captions were anonymized, and informed consent was obtained where required.

## 3. Results

### 3.1. Primary Tumor Assessment

Evaluation of primary bladder cancer lesions is most commonly based on direct visualization during cystoscopy, followed by histopathological verification obtained through biopsy or transurethral resection of the bladder (TURB). In recent years, imaging modalities, particularly magnetic resonance imaging (MRI), have gained increasing importance in the diagnostic pathway. In contrast, the usefulness of [^18^F]FDG PET/CT for detecting localized bladder cancer remains limited. This limitation is largely related to the physiological urinary excretion of [^18^F]FDG, which makes it difficult to differentiate tracer accumulation in the tumor from radioactive urine activity.

To address this issue, several methods have been proposed, including [^18^F]FDG washout, early-phase images, late post-voiding images, biphasic acquisitions, catheterization, bladder lavage, and forced diuresis [2,3,4,5]. Additional insights were provided by a study conducted by Yoon et al. [6], which showed that in early dynamic PET scans, SUVmax was significantly correlated with muscle invasiveness, histological grade, and pathological tumor size. A meta-analysis of six studies evaluated the diagnostic accuracy of [^18^F]FDG PET/CT in detecting bladder cancer lesions. The pooled sensitivity and specificity of PET or PET/CT in detecting bladder cancer were 80.0% and 84.0%, respectively [7]. Nevertheless, [^18^F]FDG PET/CT has a limited role in evaluating the primary tumor.

A few small-scale studies have explored the use of integrated PET/MRI systems for bladder cancer assessment [8,9]. This hybrid technique combines the unique advantages of both methods: the high contrast resolution and multiparametric nature of MRI together with metabolic information from PET. Hybrid PET/MRI scanners perform simultaneous spatial and temporal acquisitions, thus improving the alignment of structures between both modalities. Preliminary findings suggest that [^18^F]FDG PET/MRI may have potential value in the evaluation of bladder cancer patients; however, larger prospective studies are needed to clearly define its role in routine clinical practice.

### 3.2. Prognosis

In a prospective study including 42 patients with muscle-invasive bladder cancer, Kibel et al. assessed the diagnostic performance of preoperative [^18^F]FDG PET/CT for the detection of occult metastatic disease (nodal and/or distant). For identifying metastatic involvement, [^18^F]FDG PET/CT demonstrated a sensitivity of 70%, specificity of 94%, positive predictive value (PPV) of 78%, and negative predictive value (NPV) of 91%. These diagnostic performance metrics refer specifically to the ability of PET/CT to detect previously unrecognized metastatic lesions at the time of staging, which were subsequently correlated with clinical outcomes [10]. The median follow-up was 14.9 months. Importantly, [^18^F]FDG PET/CT identified occult metastatic disease in 17% of patients who had negative findings on conventional preoperative staging. Patients with positive [^18^F]FDG PET/CT results demonstrated significantly poorer relapse-free survival, overall survival (OS), and disease-specific survival (DSS) compared with those who were PET/CT-negative. In this study, [^18^F]FDG PET/CT findings showed a strong association with survival outcomes. In a larger two-center study, Mertens et al. evaluated 211 patients with muscle-invasive bladder cancer who underwent both [^18^F]FDG PET/CT and conventional CT for primary staging [11]. Patients were stratified into PET/CT-positive and PET/CT-negative groups, and staging information as well as mortality data were analyzed retrospectively based on prospective databases. The median follow-up was 18 months. Patients with positive PET/CT had significantly shorter OS and DSS (median OS: 14 vs. 50 months, *p* < 0.001; DSS: 16 vs. 50 months, *p* < 0.001). Multivariate analysis revealed that the presence of extravesical lesions detected on PET/CT was an independent predictor of mortality, with a hazard ratio of 3.0.

### 3.3. Staging

Clinically, approximately 30% of patients present with muscle-invasive bladder cancer [12], defined as tumor infiltration into or beyond the muscular layer of the bladder wall. Muscle-invasive bladder cancer (MIBC) is an aggressive epithelial malignancy with a high rate of early systemic metastasis. The most common sites of metastases are lymph nodes, liver, lungs, bones, and adrenal glands. Consequently, optimal therapeutic decision-making relies on precise disease staging and accurate identification of metastatic involvement [13,14].

The sensitivity of conventional imaging modalities, such as computed tomography (CT) and magnetic resonance imaging (MRI), for detecting lymph node metastases is limited because these techniques primarily rely on size-based criteria [15,16]. Thus, both methods may not detect micrometastases resulting in false negative results. Conversely, enlarged lymph nodes may reflect benign inflammatory processes, thereby reducing specificity and increasing the rate of false-positive results.

**Figure 1:**
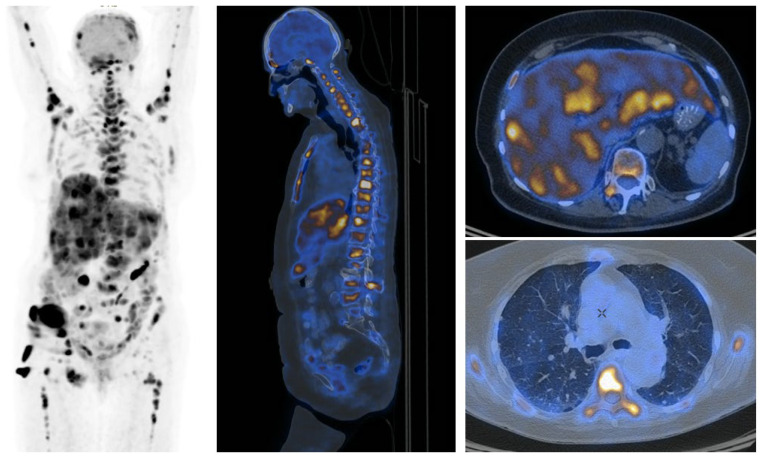
[^18^F] FDG PET/CT scan shows a 73-year-old woman with bladder cancer post-cystectomy. A dose of 4 MBq/kg was administered, and imaging was performed approximately 60 min after tracer injection. Multiple metastases are observed in the bones and liver with high [^18^F]FDG uptake. Additionally, multiple metastases, up to 8 mm in size, are detected in the lungs without tracer uptake.

Because [^18^F]FDG PET is capable of identifying increased metabolic activity in lymph nodes that are not morphologically enlarged, [^18^F]FDG PET/CT has been used in clinical trials to assess the staging of N and M features [10,17,18,19,20,21,22,23,24,25]. Studies have shown that [^18^F]FDG PET/CT detects more malignancies than conventional CT and MRI in 20–40% of patients [22,23], and it is important to note that [^18^F]FDG PET/CT can change clinical management in as many as 68% of patients [23]. Upstaging is more common than downstaging [22] (Figure 1).

Soubra et al., in a single institution study and systematic review with meta-analysis, evaluated the diagnostic accuracy of [^18^F]FDG PET/CT in detecting lymph node metastases. Eight studies met the inclusion criteria. The total sensitivity of [^18^F]FDG PET/CT in detecting lymph node metastases was 57%, while specificity reached 95%. The authors concluded that [^18^F]FDG PET/CT outperforms CT alone in identifying nodal metastases. CT alone correctly identified 22% fewer patients with lymph node metastases compared to [^18^F]FDG PET/CT [26]. These findings highlight the added value of metabolic information provided by [^18^F]FDG PET/CT, which results in more accurate staging of bladder cancer.

Similarly, Lu et al. conducted a meta-analysis assessing the role of [^18^F]FDG PET/CT in both primary staging and restaging of bladder cancer. Six studies were included [10,18,23,27,28,29], yielding a pooled sensitivity of 82%, specificity of 89%, and overall diagnostic accuracy of 92% [30]. The combined sensitivity and specificity of [^18^F]PET/CT in the detection of primary bladder cancer lesions were 90% and 100%, respectively. In the evaluation of disease stage and metastatic spread, [^18^F]FDG PET or PET/CT demonstrated pooled sensitivity and specificity values of 82% and 89%, respectively.

Vind-Kezunovic et al. investigated the prognostic value of lymph node SUVmax on [^18^F]FDG PET/CT in patients undergoing cystectomy with pelvic lymph node dissection, using histopathology as a reference standard [31]. When applying an SUVmax threshold greater than 4, the sensitivity was 61.8%, and the specificity was 84.5%. Moreover, a two-year follow-up showed that a higher SUVmax is correlated with a higher risk of recurrence, regardless of conventional pathological outcomes [31]. Thus, SUVmax values of the lymph nodes in [^18^F]FDG PET/CT may be a useful tool for the preoperative assessment of lymph node metastases.

Finally, a study by Marandino et al. demonstrated that [^18^F]FDG PET/CT may facilitate optimal patient selection among those with muscle-invasive bladder cancer who are most likely to benefit from neoadjuvant immunotherapy strategies [32].

### 3.4. Recurrence

In the case of recurrent bladder cancer, the overall prognosis is challenging. Muscle-invasive bladder cancer (MIBC) usually presents with early and predominantly distant recurrences, with nearly 50% of patients developing disease relapse after radical cystectomy. Local recurrence accounts for approximately 30% of cases, while distant metastatic spread is more frequently observed. Upon detecting a relapse, prompt action is necessary for rescue and or palliative therapy [12].

Before the initiation of further costly and potentially toxic therapies, a thorough restaging evaluation is mandatory [33]. However, data regarding the role of [^18^F]DG PET/CT in the assessment of recurrence and metastatic disease in patients previously treated for primary bladder cancer are limited. Several studies have explored the diagnostic performance of [^18^F]FDG PET/CT in this clinical setting [27,28,29,34,35].

**Figure 2:**
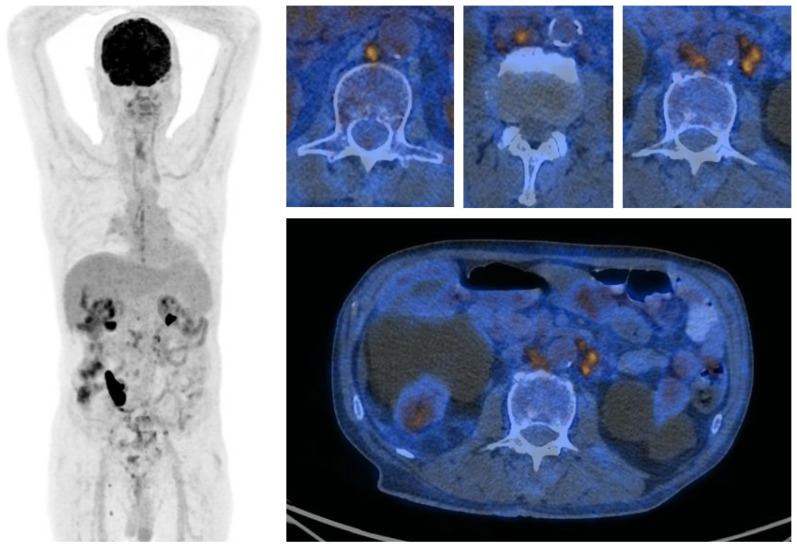
[^18^F]FDG PET/CT, a 74-year-old patient with bladder cancer after cystectomy. A dose of 4 MBq/kg was administered, and imaging was performed approximately 60 min after tracer injection. Recurrence in retroperitoneal lymph nodes with high tracer uptake. Increased tracer activity in the sternum is associated with another previous surgery.

Jadvar et al. investigated the value of [^18^F]FDG PET or PET/CT in patients with suspected recurrent or metastatic bladder cancer who had all undergone prior treatment [29]. Sites of metastases detected in the study included the mediastinum, lungs, and bones. [^18^F]FDG PET/CT altered clinical management in 17% of patients, prompting additional therapy or wait-and-see strategies (Figure 2).

Alongi et al. evaluated the accuracy, impact on treatment decisions and prognostic value of [^18^F]FDG PET/CT in patients with suspected relapse of bladder cancer [35]. [^18^F]FDG PET/CT demonstrated high diagnostic performance, with sensitivity, specificity, positive predictive value, negative predictive value, and overall accuracy of 87%, 94%, 95%, 85%, and 90%, respectively. Progression-free survival (PFS) was significantly longer in patients with negative PET/CT findings compared to those with pathological uptake (85% versus 24%). Furthermore, a normal test result was associated with longer overall survival (OS) (88% vs. 47% after two years and 87% vs. 25% after three years, respectively, *p* < 0.05). Quantitative PET parameters also demonstrated prognostic significance. The investigators demonstrated that a cut-off value of SUVmax 6 provided significant discrimination for progression-free survival. Patients with SUVmax < 6 had a 2-year PFS of 62%, compared with 15% for those with SUVmax ≥ 6, indicating a statistically significant association between higher SUVmax and worse outcomes (*p* = 0.018). Thus, higher SUVmax values served as an adverse (negative) prognostic indicator. A similar threshold was evaluated for TLG using a cut-off value of 8.5. Patients with TLG < 8.5 had a 2-year PFS of 66%, compared with 18% for those with TLG ≥ 8.5. However, this difference did not reach statistical significance (*p* = 0.09) and should therefore be interpreted as a non-significant trend toward poorer prognosis in patients with higher TLG values.

Data indicate that [^18^F]FDG PET/CT can be useful in detecting recurrent local lesions, differentiating between local recurrent disease and postoperative or post-irradiated fibrosis/necrosis, and in detecting distant metastases.

### 3.5. Response to Therapy

Patients with bladder cancer and regional lymph node metastases may benefit from neoadjuvant chemotherapy, whereas those with distant metastatic disease should be spared the unnecessary morbidity associated with radical cystectomy. Cisplatin-based neoadjuvant combination chemotherapy has been shown to improve outcomes in patients with muscle-invasive bladder cancer [36]. However, because a substantial proportion of patients do not respond to neoadjuvant treatment, early assessment of lymph node response may help in identifying candidates who are most likely to benefit from subsequent surgical intervention.

Conventional imaging modalities such as computed tomography (CT) and magnetic resonance imaging (MRI) are often suboptimal for evaluating treatment response in metastatic disease. This limitation is largely due to difficulties in distinguishing viable tumor tissue from post-therapeutic necrotic masses, as well as in detecting small metastatic deposits within lymph nodes of normal size. In this context, [^18^F]FDG PET/CT has emerged as a promising tool for assessing response to neoadjuvant chemotherapy in patients with muscle-invasive bladder cancer [37,38,39].

In the study by Mertens et al., metabolic response was evaluated in accordance with the recommendations of the European Organization for Research and Treatment of Cancer (EORTC), based on changes in [^18^F]FDG uptake observed in PET/CT [37]. Radiological response was assessed using CT according to the Response Evaluation Criteria in Solid Tumors (RECIST). All patients underwent pelvic lymph node dissection (PLND) with subsequent histopathological examination. PET/CT-based assessment of lymph node response was feasible in all cases. [^18^F]FDG PET/CT accurately differentiated responders from non-responders in 95% and 79% of cases, respectively, and correctly identified complete responders and patients with residual disease in 68% and 63% of cases, respectively. Although the small sample size precludes definitive conclusions, these findings suggest that [^18^F]FDG PET/CT may be useful for evaluating lymph node response to neoadjuvant chemotherapy and for distinguishing between responders and non-responders [37].

Van de Putte et al. evaluated the role of [^18^F]FDG PET/CT in assessing the response to neoadjuvant or induction chemotherapy for invasive bladder cancer in muscle [39]. PET/CT findings were compared with pathological results obtained after radical cystectomy, which served as the reference standard. Downstaging assessed by [^18^F]FDG PET/CT demonstrated a sensitivity of 83% and a specificity of 80%. The authors concluded that metabolic response assessment may be used to guide adjustments in neoadjuvant treatment strategies. Additionally, compared with CT alone, FDG PET/CT-based response evaluation after two cycles of first-line chemotherapy was associated with prolonged progression-free survival (PFS) and overall survival (OS) in patients with advanced bladder cancer [40].

Evidence also supports a potential role for [^18^F]FDG PET/CT in assessing response to induction chemotherapy in patients with oligometastatic bladder cancer [38]. Nevertheless, the clinical utility of [^18^F]FDG PET/CT in response assessment requires validation in larger, prospective studies before it can be fully integrated into routine clinical practice.

### 3.6. Non-FDG Radiotracers

#### 3.6.1. Choline

Studies have assessed the clinical utility of [^11^C]choline PET/CT in patients with bladder cancer [41,42,43,44,45,46,47]. The sensitivity for detecting lymph node metastases appears relatively high [41,43,44]. Brunocilla et al. evaluated the diagnostic performance of [^11^C]choline PET/CT for preoperative lymph node staging in patients eligible for radical cystectomy with extended pelvic lymph node dissection (PLND) [43]. In their analysis, [^11^C]choline PET/CT demonstrated a sensitivity of 42% and a specificity of 84%, whereas conventional CT showed a sensitivity of only 14% with a specificity of 89%.

In a lymph node-based analysis, choline PET/CT demonstrated a sensitivity of 10% and a specificity of 64%, compared to CT with a sensitivity of 2% and a specificity of 63%. These findings illustrate the variability in reported diagnostic performance and highlight the influence of study design and analytical approach. Despite these findings, other investigators have reported that [^11^C]choline PET/CT does not provide a significant diagnostic advantage over CT alone for preoperative lymph node staging in bladder cancer [45].

Nevertheless, [^11^C]choline PET/CT may have potential value in the restaging of patients with suspected bladder cancer recurrence, particularly for the evaluation of lymph node involvement and distant metastatic disease, as demonstrated by Graziani et al. [46]. A practical limitation of this tracer is its short half-life time of approximately 20.4 min, which necessitates the availability of an on-site cyclotron and restricts its widespread clinical use.

#### 3.6.2. Acetate

A prospective small-scale study evaluated MRI, PET/CT with [^11^C]acetate and CT for bladder cancer staging [48]. The results demonstrated comparable diagnostic accuracy among all three imaging modalities. It has been recognized that prior intravesical and/or systemic chemotherapy may adversely affect staging accuracy, regardless of the imaging technique used. This factor represents an important potential confounder when interpreting imaging results in heterogeneous patient cohorts.

In another study, acetate-based PET/CT was compared with choline-based PET/CT in bladder cancer [49]. Both PET/CT examinations were performed within a one-week interval, and the two radiotracers showed similar diagnostic performance in the preoperative staging setting. Overall, the clinical role of acetate-based PET/CT in bladder cancer remains limited, and current data do not support its routine use

#### 3.6.3. Methionine

Increased uptake of [^11^C]methionine in tissues reflects amino acid transport and metabolic activity, which are frequently elevated in malignant tumors. In a small study, methionine outperformed [^18^F]FDG; however, tumor detection using methionine PET alone achieved a sensitivity of only 78% [50]. Although methionine uptake was shown to correlate with tumor stage, it did not provide a notable improvement in overall bladder cancer staging.

In a separate study, methionine PET was employed to assess treatment response in bladder cancer patients at various disease stages undergoing chemotherapy [51]. PET diagnostic accuracy was poor, and the technique failed to reliably monitor therapeutic response to chemotherapy.

#### 3.6.4. Sodium Fluoride

Sodium fluoride PET/CT may offer superior detection of lytic bone metastases from bladder cancer compared with conventional [^99m^Tc]MDP bone scans. In a prospective study comparing [^18^F]NaF PET/CT with [^99m^Tc]MDP bone scans for the evaluation of skeletal metastases in bladder cancer, patients underwent both imaging modalities within a 48-h interval [52]. Fluoride PET/CT demonstrated higher diagnostic performance than planar MDP bone scintigraphy as well as MDP SPECT/CT. Fluoride PET/CT scans identified previously unrecognized bone metastases and led to changes in clinical management in approximately 35% of patients.

#### 3.6.5. Fibroblast Activation Protein Inhibitors (FAPI)

A novel group of radiotracers targeting fibroblast activation protein (FAP), referred to as FAPI tracers, has recently attracted increasing interest in nuclear medicine. In contrast to conventional tracers such as [^18^F]FDG, which reflect tumor cell glucose metabolism, FAPI PET/CT visualizes the tumor stroma, particularly cancer-associated fibroblasts (CAFs) that overexpress FAP. Fibroblast activation protein is a serine protease abundantly expressed in the reactive stroma of many epithelial malignancies [53,54], including urothelial carcinoma. Radiolabeled FAPI compounds enable stromal imaging with high tumor-to-background ratios across various cancers [54,55]. Recent development has shifted from exploratory [^68^Ga]labeled tracers toward longer-lived [^18^F]labeled compounds, which are more suitable for routine clinical use. However, despite growing interest, the current body of evidence in bladder cancer remains limited in scope and largely exploratory.

Urothelial carcinoma, particularly muscle-invasive disease, is characterized by a desmoplastic and highly reactive tumor microenvironment enriched in cancer-associated fibroblasts, including FAP-positive (fibroblast activation protein-expressing) stromal cells. FAPI accumulation corresponds to stromal remodeling and extracellular matrix activity [54]. This distinct biological targeting may help overcome some of the limitations of [^18^F]FDG PET/CT in urinary tract imaging [24]. However, the biological plausibility of this mechanism has not yet translated into robust clinical evidence demonstrating improved patient outcomes.

To directly compare tracer performance, an early intra-individual analysis was conducted in eight patients with bladder cancer who underwent both [^68^Ga]FAPI and [^18^F]FDG PET/CT. FAPI demonstrated significantly higher mean SUVmax values (8.16 vs. 4.64; *p* = 0.01) and detected approximately 30% more lesions [56], indicating superior lesion contrast and promising results for detecting pelvic lymph nodes adjacent to the urinary bladder. Nevertheless, this study was retrospective and included a very small cohort, substantially limiting statistical power and generalizability.

In a subsequent pilot study involving fifteen patients, [^68^Ga]FAPI-46 PET/CT enabled clear visualization of primary tumors, nodal involvement and distant metastases, with a total of 64 lesions identified [57]. Notably, several metastatic lesions not detected on contrast-enhanced CT were successfully visualized using FAPI PET/CT. While encouraging, these findings derive from a small cohort.

With regard to lymph node assessment, accurate nodal staging is essential for selecting patients eligible for radical cystectomy. In a 2024 histopathology-correlated study including eighteen patients who underwent [^68^Ga]FAPI-46 PET/CT, the technique correctly identified 4 of 7 metastatic lymph node regions, corresponding to a sensitivity of 57.1% and a specificity of 95.2% [58]. Although sensitivity was only moderate, the high negative predictive value (93.0%) suggests that [^68^Ga]FAPI-46 PET/CT may be useful as a rule-out imaging tool for nodal metastases. Importantly, the limited number of metastatic nodal regions reduces the robustness of these estimates.

In another study, 51 high-risk patients underwent either [^68^Ga]FAPI-46 or [^18^F]FAPI-74 PET/CT prior to cystectomy [59]. Across 123 lymph node regions, visually assessed sensitivity ranged from 55.5% for [^18^F]FAPI-74 to 63.6% for [^68^Ga]FAPI-46, while specificity exceeded 95% for both tracers. The application of quantitative SUV thresholds further enhanced diagnostic accuracy. Notably, [^18^F]FAPI-74 demonstrated performance comparable to [^68^Ga]FAPI-46, supporting its potential for broader clinical implementation.

A 2025 analysis including 34 patients with metastatic urothelial carcinoma demonstrated that [^68^Ga]FAPI-46 PET/CT detected 98% of all lesions and identified lymph node metastases not visible on CT in eight cases [60]. In a subset of patients, [^18^F]FDG PET/CT detected slightly more lesions; however, FAPI PET/CT consistently achieved higher tumor-to-background ratios across multiple metastatic sites. These findings support the use of FAPI PET/CT as a complementary imaging modality rather than a replacement for [^18^F]FDG PET/CT. Nevertheless, the retrospective design and modest cohort size limit the strength of these conclusions.

A 2024 systematic review evaluating FAPI PET/CT in genitourinary malignancies reported consistent detection of both primary and metastatic urothelial carcinoma lesions [61]. However, the authors emphasized several limitations, including urinary tracer excretion, heterogeneity among patient populations, and small study cohorts, underscoring the need for larger, well-designed multicenter trials.

The most consistent advantage of FAPI PET/CT is its high tumor-to-background contrast. Compared to [^18^F]FDG, FAPI tracers show minimal urinary retention, resulting in improved visualization of pelvic lesions, particularly primary tumors and pelvic lymph nodes [56,57,58]. Multiple studies have reported high specificity of FAPI imaging, which reflects the selective expression of FAP in tumor-associated fibroblasts. However, these specificity estimates are largely derived from small, single-center cohorts and should be interpreted cautiously, as confidence intervals are wide and external validation is limited. The development of [^18^F]labeled tracers, especially [^18^F]FAPI-74, represents an important step toward broader clinical implementation by improving tracer availability, simplifying logistics, and reducing per-scan costs [1]. Another notable advantage of FAPI PET/CT is its enhanced ability to detect CT-occult metastases, particularly within lymph nodes and the skeletal system [57,61]. However, the incremental clinical benefit of detecting additional lesions has not yet been clearly linked to improved survival or treatment outcomes.

Despite its high specificity, FAPI PET/CT demonstrates only moderate sensitivity, particularly for small lymph node metastases measuring less than 5 mm, a limitation shared with other PET/CT techniques. Although physiologic urinary excretion of FAPI tracers is lower than that of [^18^F]FDG, residual activity may still impair visualization of intraluminal or perivesical lesions in selected patients. Additionally, false-positive findings may occur due to tracer uptake in inflammatory or fibrotic tissues. Current evidence is derived from small and heterogeneous study populations, underscoring the need for standardized imaging protocols, validated SUV thresholds, and uniform interpretation criteria.

Overall, the present level of evidence supporting FAPI PET/CT in bladder cancer can be considered preliminary and hypothesis-generating rather than practice-changing. At present, FAPI PET/CT cannot replace established staging modalities but may serve as a complementary tool in cases with equivocal lymph node findings on CT or MRI, for early metastasis detection, for restaging (particularly in metastatic urothelial carcinoma) or when contrast-enhanced CT is contraindicated. Its routine integration into clinical algorithms awaits validation in large, prospective, multicenter trials with standardized methodology and outcome correlation.

### 3.7. International Guidelines

The role of [^18^F]FDG PET/CT in the assessment of muscle-invasive bladder cancer varies across international guidelines. According to the European Association of Urology (EAU) Guidelines on Muscle-invasive and Metastatic Bladder Cancer (2025 edition), PET/CT may be considered for evaluating locally advanced disease, assessing lymph node involvement, and detecting distant organ metastases. Similarly, the National Comprehensive Cancer Network (NCCN) Clinical Practice Guidelines in Oncology: Bladder Cancer (Version 2025) consider FDG PET/CT an optional adjunct imaging modality, particularly in patients with muscle-invasive disease or suspected metastatic spread.

The American Urological Association (AUA)/Society of Urologic Oncology (SUO) Guideline on Muscle-Invasive Bladder Cancer (2020; updated 2024) states that the role of PET imaging in bladder cancer staging remains uncertain and is not routinely recommended for initial staging. Although PET/CT may be useful for clarifying equivocal findings and identifying additional metastatic sites, it should not be routinely used for surveillance, as there is currently no clear evidence demonstrating superiority over conventional imaging modalities.

In the context of oligometastatic disease, both the EAU and NCCN guidelines acknowledge that advanced imaging modalities, including PET/CT, may assist in treatment planning when considering radical local therapy or metastasis-directed strategies. For restaging after systemic therapy or in cases of suspected recurrence, PET/CT may also be considered when conventional imaging findings are inconclusive. However, no major guideline currently mandates its routine use in these clinical settings.

## 4. Conclusions

PET/CT has become an important adjunct to conventional imaging in the management of bladder cancer, offering functional and molecular information beyond anatomical assessment. Although the role of [^18^F]FDG PET/CT in the evaluation of the primary bladder tumor is limited by physiological urinary excretion, substantial evidence supports its clinical value in staging, prognostic stratification, detection of recurrence, and assessment of treatment response in selected patient populations. [^18^F]FDG PET/CT has demonstrated particular utility in identifying nodal and distant metastases and in influencing therapeutic decision-making. However, its integration into routine clinical algorithms remains dependent on specific clinical scenarios and current guideline recommendations. International guidelines consistently consider FDG PET/CT a complementary rather than primary staging modality in bladder cancer.

The development of non-FDG radiotracers has further expanded the potential applications of PET/CT in bladder cancer. Tracers such as choline, acetate, methionine, and sodium fluoride have shown variable and often limited clinical benefit, whereas fibroblast activation protein inhibitor (FAPI) tracers represent a promising new approach by targeting the tumor stroma rather than tumor cell metabolism. Early clinical studies suggest that FAPI PET/CT provides high tumor-to-background ratio, high specificity, and improved detection of pelvic and CT-occult metastatic lesions, particularly in lymph nodes and bone. Despite these encouraging results, FAPI PET/CT cannot yet replace established imaging modalities but may serve as a complementary tool in selected clinical scenarios, including equivocal findings on CT or MRI, restaging of advanced disease, and cases in which contrast-enhanced imaging is contraindicated. Further large-scale, prospective, multicenter studies are required to standardize imaging protocols, validate diagnostic thresholds, and define the optimal clinical role of PET/CT and emerging radiotracers in bladder cancer management.

## Data Availability

No new data were created or analyzed in this study. Data sharing is not applicable.

## Abbreviations

The following abbreviations are used in this manuscript:

BC	Bladder Cancer
LN	Lymph Nodes
PFS	Progression Free Survival
OS	Overall Survival
DSS	Disease-Specific Survival
[18F]FDG	18F Fluorodeoxyglucose
PET/CT	Positron Emission Tomography/Computed Tomography
MIBC	Muscle-invasive bladder cancer
